# Contrast-enhanced ultrasound of renal masses in the pre-transplant setting: literature review with case highlights

**DOI:** 10.1007/s00261-024-04366-w

**Published:** 2024-06-20

**Authors:** Krister J. Barkovich, Amanda C. Gibson, Sneh Brahmbhatt, Sindhura Tadisetty, Emory C. Wilds, Leslie W. Nelson, Meera Gupta, Roberto Gedaly, Aman Khurana

**Affiliations:** 1 Department of Radiology, University of California, San Diego, La Jolla, CA 92093 USA; 2 Department of Radiology, University of Kentucky, Lexington, KY 40508 USA; 3 Department of Radiology, Mayo Clinic Florida, Jacksonville, FL 32224 USA; 4 College of Medicine, University of Kentucky, Lexington, KY 40506 USA; 5 Department of Radiology, University of Wisconsin School of Medicine and Public Health, Madison, WI 53792 USA; 6 Department of Surgery, University of Kentucky, Lexington, KY 40508 USA

**Keywords:** Ultrasonography, Contrast-enhanced ultrasound, Bosniak, Renal transplant

## Abstract

With the rising incidence of chronic kidney disease worldwide, an increasing number of patients are expected to require renal transplantation, which remains the definitive treatment of end stage renal disease. Medical imaging, primarily ultrasonography and contrast-enhanced CT and/or MRI, plays a large role in pre-transplantation assessment, especially in the characterization of lesions within the native kidneys. However, patients with CKD/ESRD often have relative contraindications to CT- and MR-contrast agents, limiting their utilization within this patient population. Contrast-enhanced ultrasound (CEUS), which combines the high temporal and spatial resolution of ultrasonography with intravascular microbubble contrast agents, provides a promising alternative. This review aims to familiarize the reader with the literature regarding the use of CEUS in the evaluation of cystic and solid renal lesions and provide case examples of its use at our institution in the pre-transplant setting.

**Graphical abstract:**

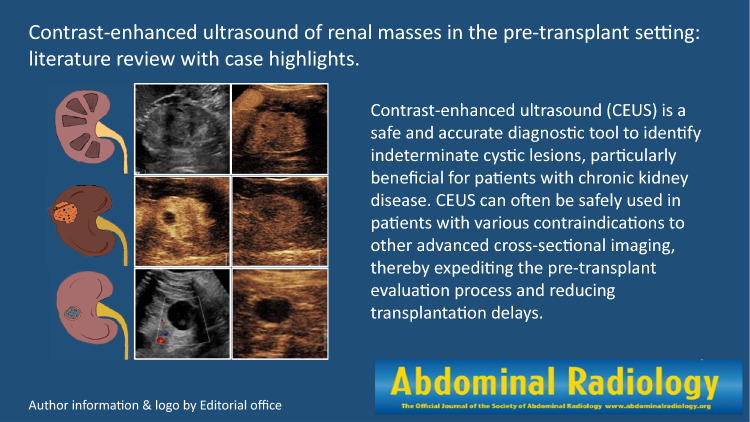

**Supplementary Information:**

The online version contains supplementary material available at 10.1007/s00261-024-04366-w.

## Introduction

Chronic kidney disease (CKD) is one of the major causes of morbidity and mortality in the United States, with an estimated 14.0% of the population having a low estimated glomerular filtration rate (eGFR), albuminuria, or both, according to the National Health and Nutrition Examination Survey (NHANES) [1, 2]. Owing to the rising rates of obesity, diabetes mellitus, and hypertension, which are well-established risk factors for the development of CKD, rates are expected to continue to rise [3]. The incidence of end-stage renal disease (ESRD), defined as severe CKD with eGFR < 15 mL/min, also continues to rise, with the total number of patients receiving kidney replacement therapy numbering over 800,000 in 2021 [1]. Healthcare expenditures for patient with ESRD in 2021 were estimated at $52.3B, accounting for about 7% of total Medicare expenditures annually. The definitive treatment for ESRD is renal transplantation, which substantially decreases adjusted mortality rates, for example to 82.8 per 1000 person-years for a 66–74 year old female, compared to 294.9 per 1000 person-years for an aged-matched patient on hemodialysis [1]. As such, there is a desire to maximize the possible number of transplant-eligible patients.

Medical imaging assumes a significant role in the assessment of renal transplant patients prior to surgical intervention since patients with conditions such as renal malignancies are deemed unsuitable candidates for urgent transplantation before the malignancy has been removed or treated. Patients with ESRD do have higher rates of cancer of the kidney, bladder, and thyroid and other endocrine organs, and ESRD is a confirmed risk factor for the development of renal cell carcinoma (RCC) [4, 5]. However, greater than 40% of sampled patients have benign simple renal cysts on CT scan and patients on long-term hemodialysis often develop acquired cystic kidney disease (ACKD) [6, 7]. Furthermore, ACKD independently increases the risk for ACKD-associated RCC [8]. Therefore, a critical question arises concerning patients with newly diagnosed solid or complex cystic lesions on pre-transplantation imaging tests.

### Medical imaging of renal masses

The mainstays of renal imaging in the pre-transplantation setting are ultrasonography (US), computed tomography (CT), and magnetic resonance (MR) imaging, each of which has its own benefits and drawbacks in terms of ease of access, cost, and sensitivity. Benign simple cystic lesions can easily be identified with classic ultrasound based on smooth well-delimited margins, an imperceptible sub-millimeter wall, and marked posterior enhancement [9]. More complex, or “atypical” cystic lesions are those that do not meet these strict criteria, and are often risk stratified using the Bosniak classification [10–12]. While this classification system was originally based on multi-phase contrast-enhanced CT (CECT), the classification system has also been expanded to include multiphase MRI [13, 14]. Multiple recent review articles summarize the use of CECT and MR imaging in the evaluation of complex cystic renal lesions [9, 15].

A key branch in the decision tree for the classification of indeterminant renal lesions is the presence or absence of contrast-enhancement; a single center study found an odds ratio of 9.7 for the detection of malignant versus benign renal lesions in the presence of renal mass septal enhancement, far greater than any other single CT feature in the prediction of malignancy [16]. However, one of the challenges in the pre-transplant evaluation of renal masses lies the safety of recipients for conventional contrast material infusion, which is often withheld in patients with compromised renal function (eGFR < 30 mL/min) [17]. Furthermore, higher rates of nephrogenic systemic fibrosis (NSF) after the administration of gadolinium-based MRI contrast agents in patients with reduced renal function, limits their use as well [18]. The majority of the patients with ACKD have simple or proteinaceous/hemorrhagic cysts with no significant risk of malignant conversion but given lack of contrast enhanced CT or MR exams (secondary to renal failure), these lesions are usually incompletely characterized, therefore resulting in delay of diagnosis and possible unnecessary surgical resection of potentially benign or indolent masses [19].

### Contrast-enhanced ultrasound

An emerging solution to the above problem is the use of contrast-enhanced ultrasound (CEUS), which combines the high temporal resolution of ultrasonography with the use of high contrast intravascular microbubbles as contrast agents [20, 21]. These ultrasound contrast agents (UCAs) are typically 2–6 µm in diameter and are composed of a biocompatible shell (lipid, protein, or phospholipid) filled with a high molecular weight and low-solubility filling gas (nitrogen, perfluorocarbon, or sulfur hexafluoride) [22]. Currently, only a single intravascular UCA, LUMASON (Bracco, NJ), is FDA approved in United States for non-cardiac use [U.S Food and Drug Administration, 2023]. Unlike iodine- and gadolinium-based contrast agents which are primarily renally cleared, the lipid surfactant of the microbubbles is excreted by hepatic metabolism and the inert gas within is exhaled, so it can be safely administered to patients with severe renal impairment [23]. Sulfur hexafluoride-based UCAs have excellent safety profiles and low rates of anaphylactoid-type reactions [24, 25]. In addition, ultrasonography does not subject the patient to ionizing radiation, can be performed in a portable setting, and owing to the short half-life of ultrasound contrast agents, multiple injections/examinations can be performed in a single day [21].

The renal cortex shows rapid enhancement after UCA administration, followed by gradual fill-in of the renal medulla, which become near isoechoic to the cortex 30 to 40 s after contrast injection [26]. The renal collecting system does not opacify because UCAs do not show renal excretion. As such, in their 2011 update, the European Federation of Societies for Ultrasound in Medicine and Biology (EFSUMB) published recommendations for the use of CEUS in the kidneys for evaluation of suspected renal vascular disorders, such as renal artery stenosis or renal ischemia, and the evaluation of cystic and solid renal masses [27]. Furthermore, CEUS has found utility in facilitating the appropriate placement of percutaneous ablation probes, which are often performed under ultrasound guidance, and can be used for the detection of residual disease in the immediate post-procedural setting [23, 28]. In the 2020 version of the ACR Appropriateness Criteria, CEUS is indicated for the evaluation of an indeterminant renal mass in all patients [29]. Herein, we perform a review of the literature regarding the use of CEUS for the evaluation of cystic and solid renal lesions, focusing on indeterminant cystic lesions, and provide a selection of diverse case examples of its use in patients at our institution in pre-transplant population.

## Methods

CEUS became available at the University of Kentucky in 2020 and has since become increasingly utilized by transplant surgeons to characterize indeterminate renal lesions identified on noncontrast CT and MRI during pre-transplant workup. Case examples with accompanying illustrations of papillary renal cell carcinoma (RCC), clear cell RCC, Bosniak II renal cyst, hemorrhagic cyst, simple cyst and prominent column of Bertin in pre-transplant patients are presented.

## Discussion

### Contrast-enhanced ultrasound for the evaluation of solid renal lesions

**Fig. 1:**
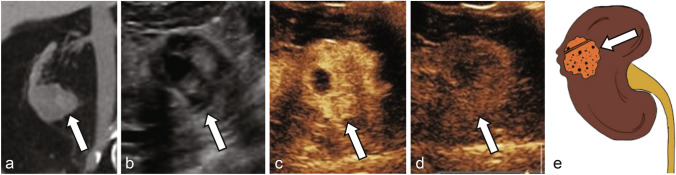
**a** Noncontrast CT shows an indeterminate lesion arising from the lower pole right kidney (arrow). Grayscale ultrasound **b** shows a mixed cystic and solid mass (arrow). CEUS shows enhancement in the early arterial phase at 27 s (**c**) with washout at 2 min (**d**), consistent with renal cell carcinoma (arrows), later pathologically proven as clear cell renal cell carcinoma. **e** Illustration of a mixed cystic and solid clear cell renal cell carcinoma (arrow)

Studies using CEUS in the evaluation of solid renal lesions have focused on defining imaging parameters to distinguish benign lesions, such as renal angiomyolipoma (AML) and oncocytoma, from those with malignant potential such as clear-cell renal cell carcinoma (ccRCC) and papillary RCC (pRCC). Tamai et al. evaluated 29 patients with solid renal tumors detected on conventional ultrasound and found 17 out of 18 ccRCC showed hypervascularity, defined as contrast enhancement greater than surrounding renal parenchyma more than 30 s after UCA injection [30]. However, oncocytoma and AMLs also showed similar hypervascularity. To better distinguish between these benign and malignant entities, Fan et al. evaluated 72 solid renal parenchyma lesions and qualitatively classified the enhancement pattern as homogeneous or heterogenous. The authors found that benign lesions such as AMLs or oncocytomas showed high rates of homogenous enhancement (18/24 lesions) while malignant ccRCCs and papillary RCCs were more likely to demonstrate heterogenous enhancement (64% of ccRCC) [31]. Similarly, in a in a prospective evaluation of 51 solid renal masses, Zhou et al. observed 63.6% of RCCs demonstrated diffuse heterogenous arterial phase (10–40 s after UCA injection) enhancement, compared to only 6.9% of AMLs [32]. Fan et al. also identified that late hyperenhancement, defined as mass enhancement greater than that of the surrounding renal parenchyma 45–55 s after UCA injection, was present in 87.3% of ccRCC and only 4.2% of AML. Gerst et al. observed a similar phenomenon, which the authors described as delayed contrast washout more than 30 s after UCA administration, in 52% of ccRCC compared to only 18% of benign or low-grade tumors. In a retrospective comparison of the enhancement characteristics of 93 RCCs and 33 renal AMLs, Xu et al. found 81% of RCCs were more often hypoenhancing relative to the renal parenchyma on corticomedullary phase (36–120 s after UCA injection) as compared to AMLs (21%), which were typically isoenhancing [33]. Similarly, Chen et al. identified that 78% of RCCs washout faster than the surrounding renal parenchyma (41–180 s after UCA injection) as compared to AMLs, which show synchronous or slower washout in 19/21 (90%) of cases [34]. Taken together, these results identify that ccRCC are more likely to show heterogenous arterial phase hyperenhancement in CEUS with pronounced washout relative to the surrounding renal parenchyma (Fig. 1), in contrast to benign hyperenhancing lesions such as AML, which show more homogenous enhancement and less pronounced washout on late phase imaging (Supplemental Fig. 1) [33, 35]. Barr et al. noted that these parameters can help distinguish between echogenic RCCs (eRCC) and AMLs, which are challenging to differentiate on non-enhanced ultrasonography, since eRCCs display arterial phase hyperenhancement with washout in late phase, while AMLs do not [33, 36].

**Fig. 2:**
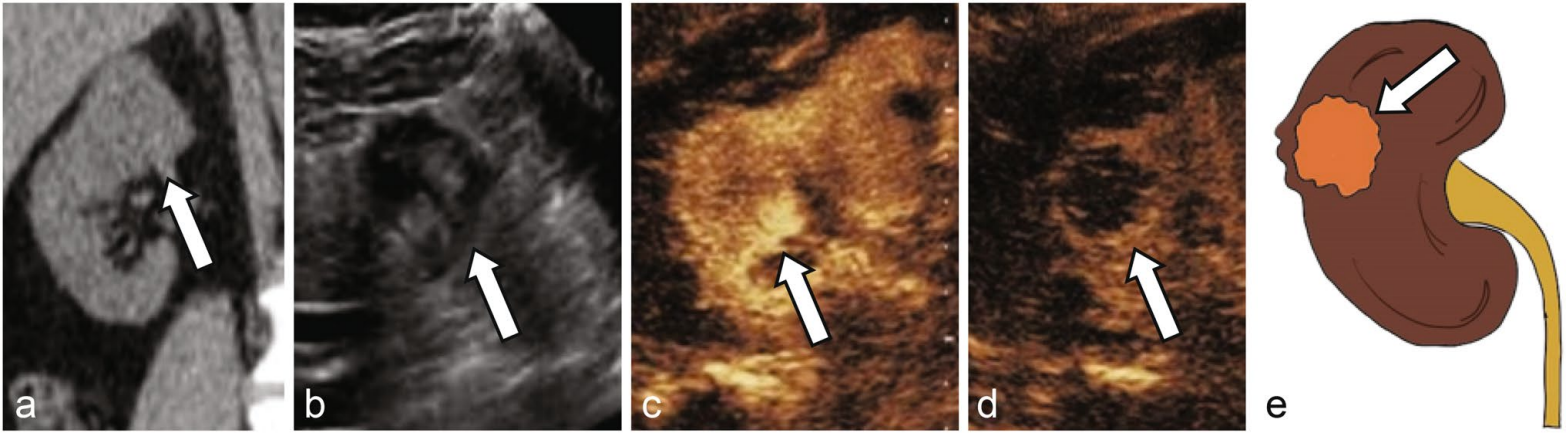
**a** Noncontrast CT shows an indeterminate lesion arising from the superior pole right kidney (arrow). Grayscale ultrasound **b** shows a solid mass with mixed echogenicity (arrow). CEUS shows enhancement in the early arterial phase at 17 s (**c**) with washout at 2 min (**d**), consistent with renal cell carcinoma (arrows), later pathologically proven as papillary renal cell carcinoma. **e** Illustration of a mostly solid papillary renal cell carcinoma (arrow)

Papillary RCC, which makes up 10–15% of RCCs, is the most common type of “nonconventional RCC” which display different imaging characteristics as compared to the more common ccRCC [37, 38]. Papillary RCCs more commonly appear hypovascular and homogenous on imaging studies [38]. On CEUS, papillary RCC are less likely to show cortical phase hyperenhancement and typically remain hypoenhancing to the renal cortex on all phases (Fig. 2) [33, 39, 40]. Chromophobe RCC, which makes up 4–6% of RCCs, are associated with a spoke-wheel like pattern of enhancement, similar to that of renal oncocytomas [41]. CEUS data of chRCC lesions is limited, but the three lesions of this subtype characterized by Xu et al. and Fan et al. all displayed cortical phase hyperenhancement with late phase washout [31, 33]. Renal oncocytoma (RO), a benign renal epithelial tumor that accounts for approximately 5% of all primary renal neoplasms, is classically characterized by a central scar on imaging [42, 43]. CEUS findings in RO are variable; for example, in a study of 13 pathologically proven ROs, Schwarze et al. found 85% (11/13) displayed cortical phase (8–35 s after UCA injection) hyperenhancement with variable (50%) washout on later phases, whereas in a study of 23 ROs, Tufano et al. identified 91% of ROs displayed hyperenhancement during cortical phase (10–45 s after UCA injection) and 87% showed synchronous/slow corticomedullary phase washout [44, 45]. Clearly, there is variability and considerable overlap in the CEUS features of these atypical RCCs and benign entities, and additional imaging tests or pathology are required for clearer discrimination [39]. CEUS characteristics of common renal lesions are summarized in Table 1.

**Table 1:** Overview of CEUS imaging features of common solid renal masses, adapted from [35]

Solid lesion	Conventional (B-mode) ultrasound findings	CEUS findings
Clear cell renal cell carcinoma (ccRCC)	- Small tumors often echogenic - Large tumors heterogeneously hypoechoic, hemorrhage and calcifications common	- Heterogenous hyperenhancing on cortical phase - Hypoenhancement (“wash-out”) on corticomedullary and delayed phases - Perilesion rim-like enhancement (“pseudocapsule”)
Papillary renal cell carcinoma (pRCC)	- Hypoechoic; internal solid components	- Homogenous - Iso/hypoenhancement relative to renal parenchyma throughout exam
Chromophobe renal cell carcinoma (chRCC)	- Variable	- Variable
Angiomyolipoma (AML)	- Homogenously hyperechoic; lipid-poor AMLs can be isoechoic	- Homogenous enhancement - Prolonged enhancement, ± slow washout
Renal oncocytoma (RO)	- Variable	- Variable
Pseudotumor	- Isoechoic	- Isoenhancing relative to renal parenchyma throughout exam

A meta-analysis of CEUS in the characterization of solid lesions found a sensitivity and specificity of 98% and 78%, respectively, for malignancy [46]. The lower specificity is almost entirely due to the false classification of benign lesions as malignant [47]. As such the negative predictive value (the likelihood of a lesion being benign if classified as benign) of CEUS approaches 100% [46].

### Contrast-enhanced ultrasound for the evaluation of complex cystic renal lesions

**Fig. 3:**
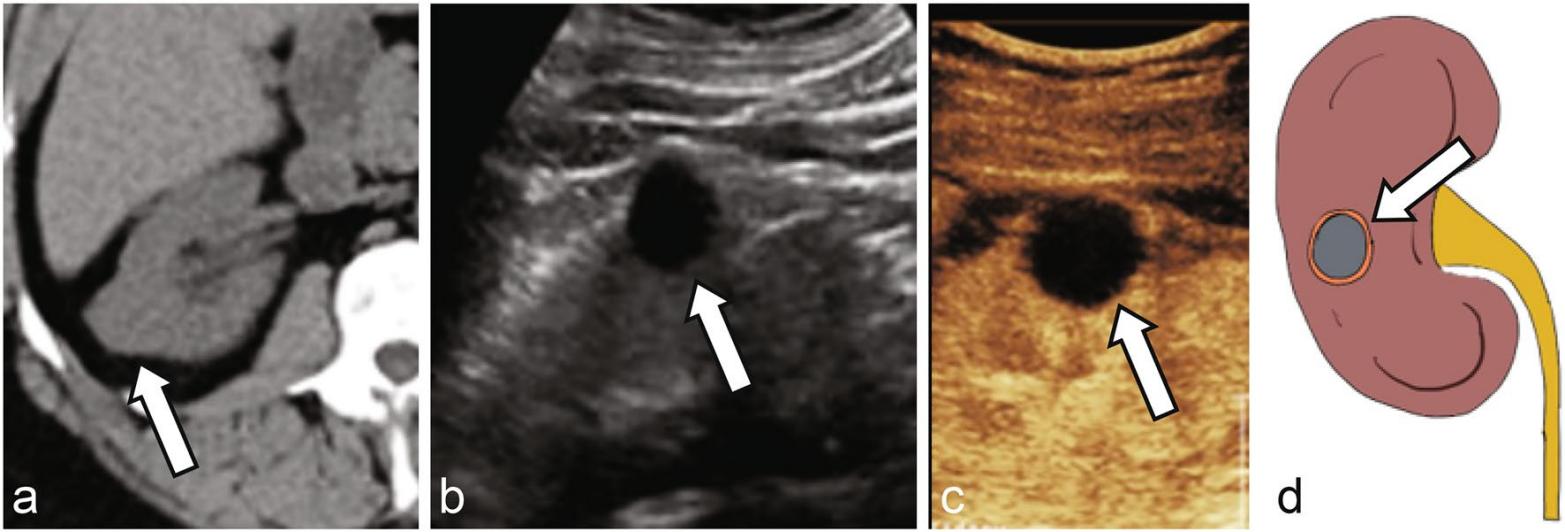
**a** Noncontrast CT shows an indeterminate lesion arising from the right kidney (arrow). Grayscale ultrasound **b** reveals an anechoic cyst with imperceptible walls and posterior acoustic enhancement (arrow). CEUS **c** confirms no enhancement in the early arterial phase (arrow), consistent with a simple cyst. **d** Illustration of a simple cyst without septation or internal debris (arrow)

**Fig. 4:**
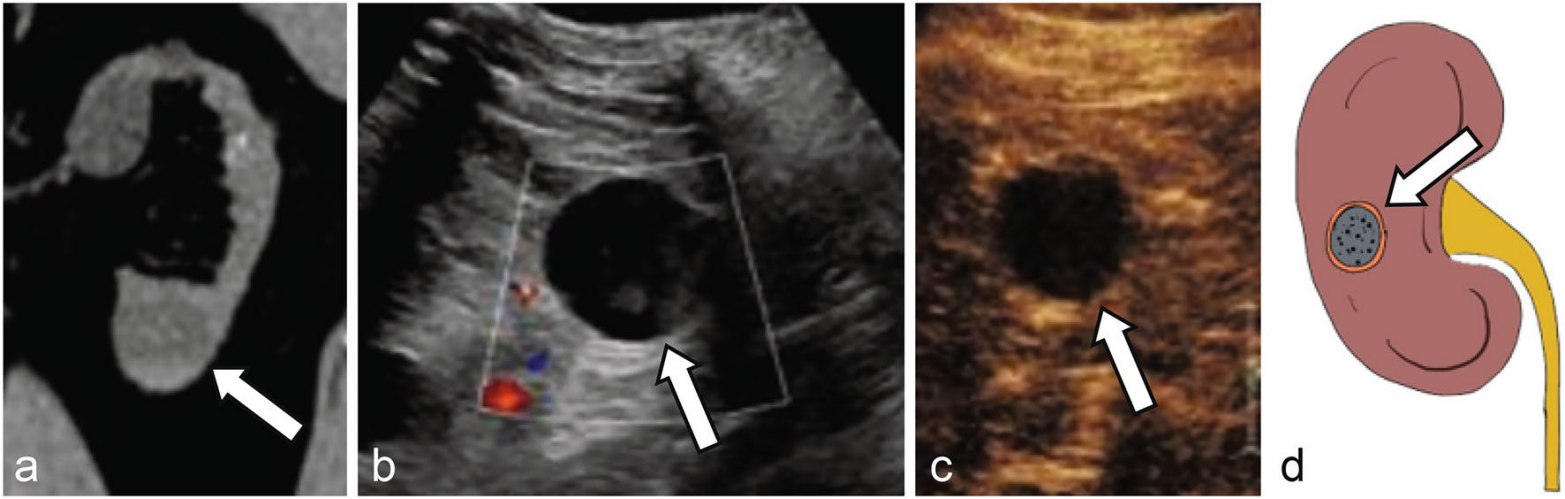
**a** Noncontrast CT shows an indeterminate lesion arising from the lower pole left kidney (arrow). Grayscale ultrasound **b** reveals a well-circumscribed cystic lesion with echogenic debris or soft tissue (arrow). CEUS **c** confirms no enhancement of the debris (arrow), consistent with a hemorrhagic cyst. **d** Illustration of a hemorrhagic cyst with internal debris (arrow)

**Fig. 5:**
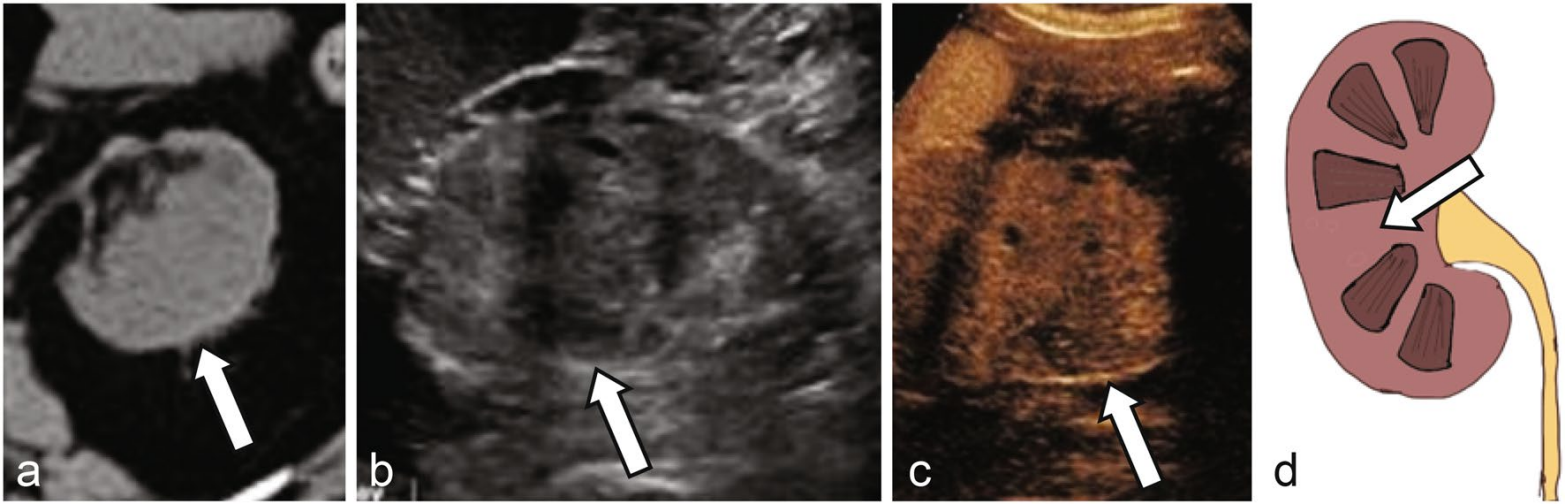
**a** Noncontrast CT shows a mass-like lobulation within the interpolar left kidney (arrow). Grayscale ultrasound **b** reveals a prominent lobulation with similar echogenicity to the adjacent renal cortex (arrow). CEUS **c** shows enhancement of this region matching the adjacent renal cortex (arrow), consistent with a prominent column of Bertin. **d** Illustration of prominent column of Bertin in continuity with adjacent renal cortex (arrow)

As with the Bosniak classification using CT and MRI, the stratification of complex cystic renal lesions based on likelihood of malignancy using CEUS is very appealing to radiologists and urologists alike [48]. When encountering an indeterminate cyst on noncontrast CT, follow up grayscale US may or may not show typical US features of a simple cyst such as anechoic lesion with thin imperceptible wall and posterior acoustic enhancement (Fig. 3). CEUS at the same encounter can easily confirm presence of simple or hemorrhagic cyst with no microbubble enhancement within the lesion, even if there is echogenic debris on grayscale US in the cases of hemorrhagic or proteinaceous cysts (Figs. 3, 4). Sometimes, CEUS helps with better evaluation of the corticomedullary differentiation and evaluation of normal anatomical variants such as prominent column of Bertin (Fig. 5), dromedary hump, fetal lobulation, etc., along with focal areas of renal scarring.

Multiple studies have confirmed the high diagnostic accuracy of CEUS in the categorization of complex cystic renal lesions [36, 47, 49–63]. Meta-analyses performed by Richard Barr and Zhou et al. found pooled sensitivities and specificities of 95% and 84%, respectively [46, 64]. These results are comparable to CECT and MRI [53, 63–65]. For indeterminant lesions (Bosniak category IIF and III), which are typically the most difficult to assess, Angelini et al. found that CEUS showed a sensitivity of 80%, specificity of 69%, PPV of 67%, and NPV of 82%, which is similar to that of CECT. A subgroup analysis showed a slight decreased in the performance of CEUS in superficial versus deep renal lesions, with area below the ROC curve (AUC; a marker of overall diagnostic accuracy) 0.84 vs 0.77, respectively [66].

**Fig. 6:**
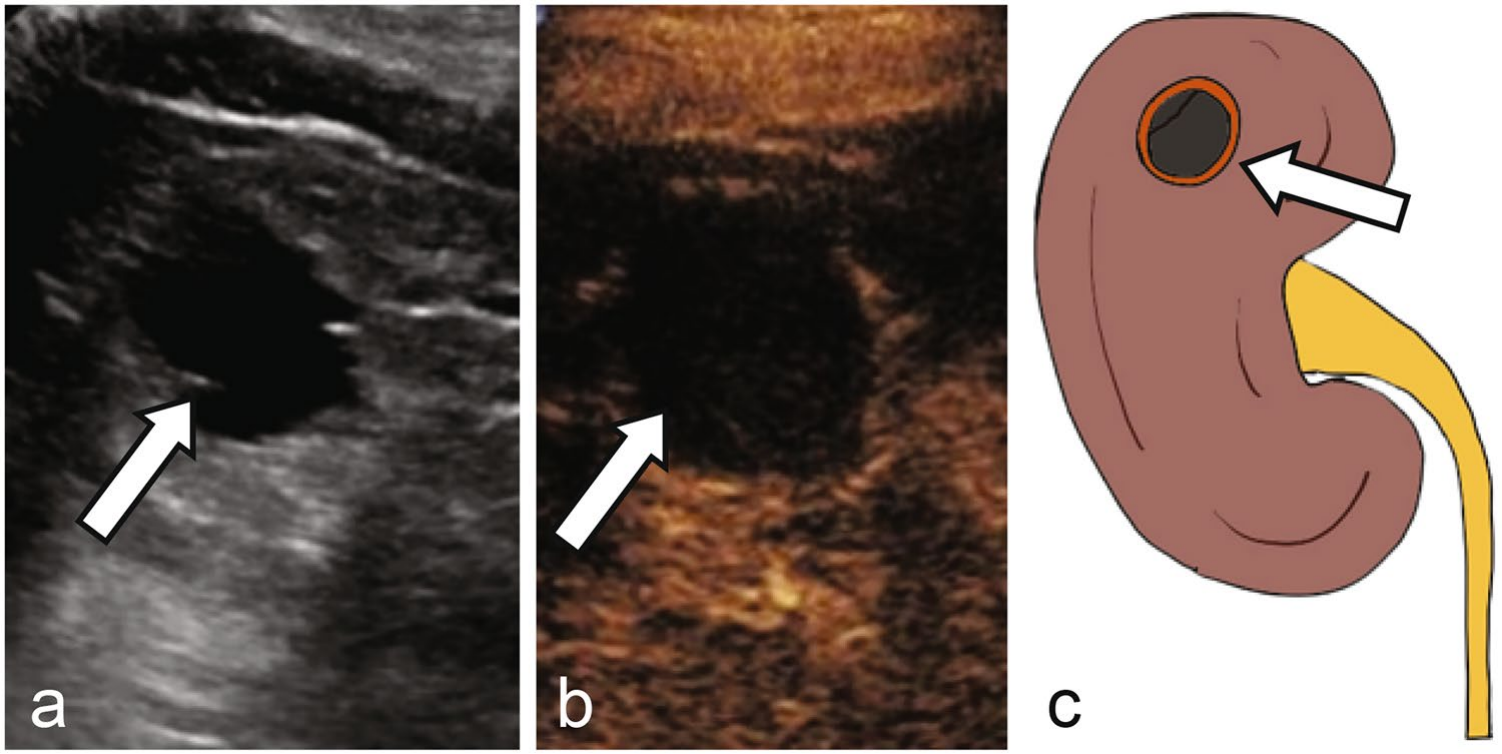
**a** Grayscale ultrasound reveals a well-circumscribed cystic lesion with an incomplete, thin septation (arrow). CEUS **b** confirms no enhancement of the thin septation (arrow), consistent with a minimally complex cyst (Bosniak II). **c** Illustration of a minimally complex cyst with a single thin septation (arrow)

A major branchpoint in the Bosniak classification system is the presence of measurable enhancement within the walls or septa of cystic renal lesions [67]. Because of the high spatial resolution of ultrasonography, it is hypothesized that CEUS has the potential to have greater sensitivity for septal or wall enhancement relative to CECT (Fig. 6). In their retrospective analysis of 31 pathologically confirmed cystic lesions characterized by CEUS and CECT, Park et al. identified that all differences in the Bosniak classification between imaging modalities were the result of upgrading by CEUS, which they hypothesized were due to improved visualization of enhancement and/or more thickened septa [50]. Similarly, in a prospective study of 44 complex cystic lesions performed by Ascenti et al., six (14%) of lesions were upgraded on CEUS due to an increased number of intracystic septa observed on CEUS, and four (9%) showed septal enhancement [49]. In a study by Sanz et al., seven lesions were classified as malignant by CEUS and not by CT, and of these seven lesions, six were malignant on histopathology [56]. Wei et al. identified improved diagnostic performance of CEUS with regards to papillary renal cell carcinoma, correctly diagnosing 13/13 lesions, compared to 8/13 by CECT (p < 0.05) [59].

However, at the expense of this increased sensitivity, Quaia et al. found CEUS mis-classified several benign cystic lesions owing to peripheral wall enhancement and thickened septal walls [51]. Similarly, Herms et al. noted other mismatches in Bosniak classification between CEUS and MRI in 22/52 cases, with all but one case resulting in higher staging by CEUS [68]. Of these, 17 were malignant on final pathology, but four were benign lesions (simple renal cysts, mixed epithelial stroma tumor (MEST)). As such, CEUS has a high negative predictive value for malignancy (approaching 100%), but may be more prone to the mis-classification of benign lesions due to lower specificity [46, 69]. A large prospective study with histopathological correlation may assist in developing a dedicated classification system based on CEUS and will likely aid in establishing which lesions can be safely observed.

### Contrast-enhanced ultrasound for the evaluation of renal lesions in patients with contraindications to CT contrast agents

A clear indication for CEUS is in the evaluation of renal lesions in patients with relative or absolution contraindications to CT- or MR- contrast agents, for example those with CKD or ESRD [29]. However, there have been limited studies that assess the clinical performance of CEUS in the evaluation of renal lesions within this patient population. Paudice et al. performed a prospective assessment of the use of CEUS in 15 renal transplant recipients diagnosed with ACKD with suspicious or nondiagnostic ultrasound [70]. They identified 27 Bosniak category I lesions, four category II, two category III, which showed enhancement of thickened septae, and two solid enhancing lesions. The Bosniak category III and solid lesions underwent surgical resection, which revealed three RCC and one papillary carcinoma. In a prospective cohort analysis of 35 patients, the majority with renal failure, who underwent a non-enhanced CT (NECT) and CEUS for evaluation of a renal lesion, Sawhney et al. found increased sensitivity in the identification of pathology (100%) relative to NECT (89%) [71]. These studies confirmed the utility of CEUS in characterization of renal lesions in patients with relative contraindication to CT contrast agents. In a prospective analysis of 19 solid renal lesions in patients on hemodialysis, Hashimoto et al. found that CEUS allowed for accurate diagnosis in 17/19 lesions, with 14 lesions identified as RCC and three as simple cysts [72]. There was one false positive of an inflammatory cyst with hyper-enhancement, and one false negative due to deep location of the lesion, both very plausible situations for false positive calls on CEUS.

To directly compare the clinical performance of CEUS in patients with differing stage of CKD, Chang et al. performed subgroup analysis of the performance of CEUS in the detection of renal malignancy patients with advanced (stage IV, V, or ESRD) versus early (stage II or III) CKD [60]. The authors found decreased overall accuracy in advanced CKD, largely due to a decreased in specificity. They hypothesized that this was due to more heterogenous enhancement of the uninvolved renal parenchyma, which attenuated the difference between the lesion and surrounding tissue. These results were not duplicated in a subsequent study, in which sensitivity and specificity for benign and malignant renal lesions were not significantly different between early and advanced CKD [73].

## Conclusion

The definitive treatment of ESRD is renal transplant. Patients with CKD and ESRD have higher rates of benign and malignant cystic renal disease, which must be fully characterized to exclude malignancy prior to eligibility for transplantation. Commonly used diagnostic imaging techniques include contrast-enhanced CT and MR imaging, both of which utilize nephrotoxic contrast agents which are relatively contraindicated in patients with CKD. CEUS provides a promising alternative, as it is not nephrotoxic and demonstrates high diagnostic performance in distinguishing benign and malignant renal lesions, with negative predictive values for malignancy approaching 100%. Renal CEUS has become increasingly utilized by transplant surgeons to characterize indeterminate renal lesions identified on noncontrast CT and MRI during pre-transplant workup. However, there is limited data for the diagnostic performance of CEUS in patients with severe CKD and ESRD. Further prospective multicenter studies are required to further establish grading criteria, perhaps a Bosniak equivalent, for the accurate and reproducible use of CEUS within this patient population.

## Supplementary Information

Below is the link to the electronic supplementary material.

Supplementary file1 (DOCX 851 KB)
