# Supplementary material for: Contrast-enhanced ultrasound of renal masses in the pre-transplant setting: literature review with case highlights
Source: Abdom Radiol (NY). 2024 Jun 20;49(12):4521–30. doi: 10.1007/s00261-024-04366-w (PMC11522065; doi:10.1007/s00261-024-04366-w)
Supplement: Supplementary file 1 — Supplementary file1 (DOCX 851 KB) [file 261_2024_4366_MOESM1_ESM.docx]

Supplemental Information for

**Contrast-enhanced ultrasound of renal masses in the pre-transplant setting: literature review with case highlights.**


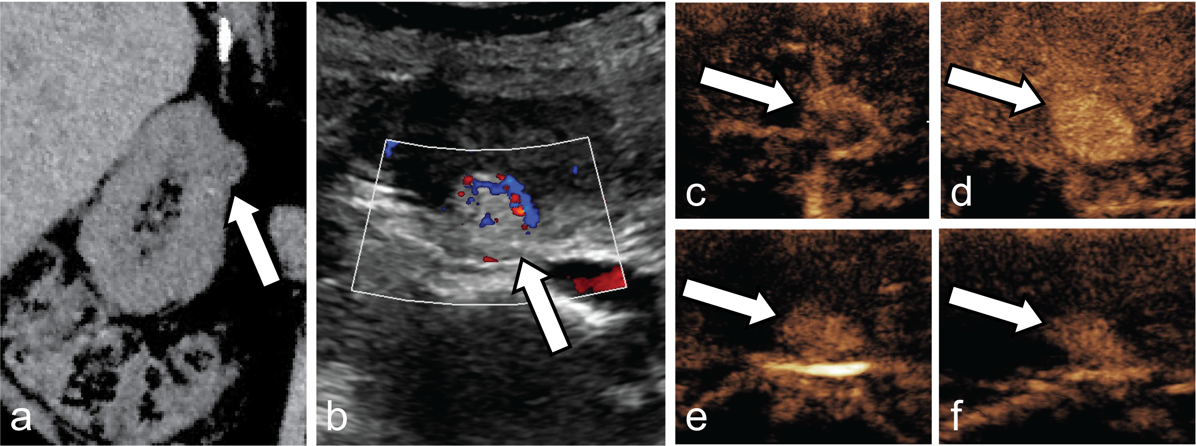


**Supp. Fig. 1.** (a) Noncontrast CT shows an indeterminate hypoattenuating lesion arising from the right kidney (arrow). Grayscale ultrasound (b) shows a well-circumscribed echogenic lesion with internal vascularity (arrow). CEUS shows early enhancement in this lesion in the early arterial phase at 10 and 19 seconds (c, d) with slow washout but persistent hyperenhancement relative to the renal parenchyma after 3 and 5 minutes (e, f), consistent with an angiomyolipoma (AML).
